# In Vitro Pharmacokinetic/Pharmacodynamic Modelling and Simulation of Amphotericin B against *Candida auris*

**DOI:** 10.3390/pharmaceutics13111767

**Published:** 2021-10-22

**Authors:** Unai Caballero, Elena Eraso, Javier Pemán, Guillermo Quindós, Valvanera Vozmediano, Stephan Schmidt, Nerea Jauregizar

**Affiliations:** 1 Department of Pharmacology, Faculty of Medicine and Nursing, University of the Basque Country (UPV/EHU), 48940 Leioa, Spain; unai.caballero@ehu.eus; 2 Department of Immunology, Microbiology and Parasitology, Faculty of Medicine and Nursing, University of the Basque Country (UPV/EHU), 48940 Leioa, Spain; elena.eraso@ehu.eus (E.E.); guillermo.quindos@ehu.eus (G.Q.); 3 Microbiology Department, Hospital Universitario y Politécnico de La Fe, 46026 Valencia, Spain; javier.peman@gmail.com; 4 Severe Infection Research Group, Health Research Institute Hospital La Fe, 46026 Valencia, Spain; 5 Center for Pharmacometrics and Systems Pharmacology, Department of Pharmaceutics, College of Pharmacy, University of Florida, Orlando, FL 32827, USA; valva@cop.ufl.edu (V.V.); SSchmidt@cop.ufl.edu (S.S.)

**Keywords:** *Candida auris*, PK/PD model, amphotericin B, time-kill curves

## Abstract

The aims of this study were to characterize the antifungal activity of amphotericin B against *Candida auris* in a static in vitro system and to evaluate different dosing schedules and MIC scenarios by means of semi-mechanistic pharmacokinetic/pharmacodynamic (PK/PD) modelling and simulation. A two-compartment model consisting of a drug-susceptible and a drug-resistant subpopulation successfully characterized the time-kill data and a modified E_max_ sigmoidal model best described the effect of the drug. The model incorporated growth rate constants for both subpopulations, a death rate constant and a transfer constant between both compartments. Additionally, the model included a parameter to account for the delay in growth in the absence or presence of the drug. Amphotericin B displayed a concentration-dependent fungicidal activity. The developed PK/PD model was able to characterize properly the antifungal activity of amphotericin B against *C. auris*. Finally, simulation analysis revealed that none of the simulated standard dosing scenarios of 0.6, 1 and 1.5 mg/kg/day over a week treatment showed successful activity against *C. auris* infection. Simulations also pointed out that an MIC of 1 mg/L would be linked to treatment failure for *C. auris* invasive infections and therefore, the resistance rate to amphotericin B may be higher than previously reported.

## 1. Introduction

*Candida auris* is a multidrug-resistant fungal pathogen that has emerged globally as a cause of different infections, such as severe cases of fungemia [1,2]. Candidemia due to this pathogen is associated with a high rate of mortality, especially in immunocompromised patients. Other risk factors for *C. auris* candidemia include previous exposure to antibiotics and underlying diseases such as diabetes, cardiovascular diseases or COVID-19 [3,4].

Additionally, the virulence and pathogenic capacity of *C. auris* and the decreased susceptibility to antifungal drugs is greatly worrying. Tentative epidemiological breakpoints for available antifungal drugs have recently been published. Those reports highlight that *C. auris* has high MIC values for polyenes, azoles, echinocandins and nucleoside analogues [5,6]. However, MIC related susceptibility categorization of *C. auris* isolates should be cautiously interpreted, since species-specific clinical breakpoints have not yet been defined [7]. *C. auris* is resistant to fluconazole and both intrinsic and acquired resistance has been reported [5,8]. Reduced susceptibility to the other azoles, including the newest isavuconazole, has also been described [8]. Echinocandins are the first line treatment to treat *C. auris* infections [9], but resistance to these drugs or therapeutic failures can emerge rapidly in *C. auris* [10].

Regarding amphotericin B, a wide range of MIC values has been reported, with resistance rates ranging from 0 to 30% using 1 mg/L as cut-off [7,11,12,13,14,15]. Recently, amphotericin B was described as the only in vitro fungicidal agent against *C. auris*, unlike echinocandins [16]. These facts, alongside with the fact that amphotericin B is the first alternative to echinocandins for *C. auris* infections [17,18], make it an interesting drug whose activity against this pathogen needs to be studied in deep.

In the current worrying scenario of reduced effective treatments to deal with *C. auris* infections, in vitro studies that use time–kill (T-K) curve experiments and pharmacokinetic/pharmacodynamic (PK/PD) models to simulate different dosing schedules and activity profiles, offer an attractive tool to describe the observed antifungal activity and to predict the efficacy of the studied drugs. There are few PK/PD models from in vitro kinetic data developed for antifungal drugs and *Candida*: caspofungin and fluconazole against *Candida albicans* [19]; voriconazole against *Candida* spp. [20]; and recently, anidulafungin against *Candida* spp. [21]. However, despite the relevance of *C. auris*, PK/PD modelling of antifungal drugs for this emergent species is still lacking.

The aim of this study was to develop a semi-mechanistic PK/PD model for amphotericin B against *C. auris* that can (a) describe the in vitro T-K experiment of clinical isolates of *C. auris* exposed to amphotericin B and (b) simulate the expected T-K curves for different dosing regimens and MIC scenarios.

## 2. Materials and Methods

Six *C. auris* blood isolates from the outbreak in Hospital Universitario y Politécnico La Fe (Valencia, Spain) were included in this study [22]. The MIC, defined as the minimum concentration producing ≥90% growth reduction, was determined following EUCAST guidelines [23]. The MIC of amphotericin B for the six isolates was 1 mg/L.

Amphotericin B was obtained from Sigma-Aldrich (Madrid, Spain) as a powder. Stock solutions were prepared with DMSO as solvent and stored at −80 °C until use.

Static T-K curve experiments were carried out on flat-bottomed microtitre plates in RPMI medium (Sigma-Aldrich), with a final volume of 200 µL per well at 37 °C for 48 h. *C. auris* blood isolates were grown at 37 °C for 24 h prior to the start of the experiment to obtain fungal cultures in early logarithmic phase growth. Cells were suspended in sterile distilled water to achieve a starting inoculum size of 1–5 × 10^5^ colony forming units (CFU)/mL and added to the microtitre plate containing amphotericin B at concentrations 0.25, 0.5, 1, 2 and 4 times the MIC. Growth control was also measured by adding the inoculum to wells containing RPMI medium without amphotericin B. Sample for viable counts were taken at 0, 2, 4, 6, 8, 24 and 48 h, plated in triplicate onto Sabouraud dextrose agar (SDA) and incubated for 24–48 h at 37 °C. Depending on drug concentration, samples were either first diluted in PBS or plated directly. When it was expected a sterilizing activity, the whole well was sampled onto an SDA plate. Experiments were performed in duplicate for each isolate on different days. The lower limit of detection was 5 CFU/mL. However, due to the well-known sterilizing activity of amphotericin B, all the samples that showed no growth at all were considered to be 0 CFU/mL. Carryover effect was determined as previously described [24].

The basis of the semi-mechanistic model included two fungal stages in the PD part of the model, consisting of a drug-susceptible fungal subpopulation (S) and a drug-resistant subpopulation (R) [25]. This two-subpopulation model accounted for the biphasic killing behaviour observed in the individual isolate static T-K curves (individual plots not shown).

First-rate order constants that defined both populations were the natural growth rate (k_growth_), natural death rate (k_death_) and the transfer constant from S into R (k_SR_). The equation that described S subpopulation in the absence of drug was as follows:

dS/dt = k_growthS_ × S × (1 − e^−αt^) − k_death_ × S − k_SR_ × S (1)

where dS/dt is the change in the number of the S subpopulation as a function of time.

It was not possible to perform a simultaneous estimation of both k_growthS_ and k_death_ in this experimental setting. Hence, in an initial fit, k_growthS_ was estimated by fitting a single-stage model [19] to the control data. Based on this estimation of k_growth_ (0.118 h^−1^) and on previous analysis, k_death_ was then fixed to 0.01 h^−1^ for final parameter estimation in the two-stage model. Parameter α accounted for the delay in growth observed due to experimental settings.

A specific k_growth_ was estimated for the R subpopulation (k_growthR_) to account for the regrowth observed at certain concentrations from 24 to 48 h. The k_death_ parameter was negligible in the final equation describing R subpopulation, hence it was not considered in the following equation:

dR/dt = k_growthR_ × R + k_SR_ × S (2)

As previously mentioned, k_SR_ is the parameter that described the transfer of fungal cells from a susceptible state into a resistant one. It was defined as follows:

(3) $$k_{\mathrm{SR}}=\frac{\begin{array}{c} \left(k_{\mathrm{growth}}-k_{\mathrm{death}})\times (S+R\right) \end{array}}{N_{\max}}$$

where S and R are the compartments with susceptible and resistant fungal populations, respectively, and N_max_ is the maximum total density of fungal population in the stationary phase (in log CFU/mL).

The effect of amphotericin B on the fungal killing of the susceptible subpopulation was modelled using an E_max_ sigmoidal equation:

(4) $$\mathrm{Drug}\;\mathrm{effect}=\frac{E_{\max}\times C^{h}}{{\mathrm{EC}}_{50}^{h}+C^{h}}$$

where E_max_ is the maximum achievable drug-induced fungal killing-rate constant, EC_50_ is the drug concentration necessary to achieve half the maximum effect, C is the drug concentration and h is a Hill factor or sigmoidicity factor that modifies the steepness of the slope and smoothens the curve.

The final model for the S and R subpopulations were described according to Equations (2) and (5):

dS/dt = k_growthS_ × S ×(1 − e^−αt^) − Drug effect × S − k_death_ × S − k_SR_ × S (5)

dR/dt = k_growthR_ × R + k_SR_ × S

All T-K data were transformed into log CFU/mL and simultaneously analysed in NONMEM v7.4 with ADVAN13 subroutine and first-order conditional estimation method (FOCE). Residual variability was estimated by using an additive model. As six clinical isolates were analysed, inter-individual variability (IIV) was checked. Additionally, inter-occasion variability (IOV) was also investigated to account for the variability that might have arisen either from each experimental day or from microtitre plate batch preparation. Model performance was assessed by precision of parameter estimates, changes in objective function value (OFV) and evaluation of diagnostic plots. Final model selection was also assisted by the performance of visual predictive checks (VPCs) and non-parametric bootstrap. VPCs were performed and graphically represented with NONMEM and S-PLUS software, stratified by concentration, with the experimental plots overlaid by the median and 95% prediction interval of a simulated virtual population of 1000 individuals. Non-parametric bootstrap was conducted by resampling 1000 datasets using Perl speaks NONMEM (PsN).

In vivo PK parameters for amphotericin B deoxycholate were extracted from a tricompartmental model previously described in the literature, V_1_ = 0.136 L/kg; V_2_ = 0.275 L/kg; V_3_ = 1.4 L/kg; Cl = 0.013 L/h/kg; Q_12_ = 0.35 L/h/kg; and Q_13_ = 0.026 L/h/kg [26]. The effect of treatments with standard clinical doses of 0.6, 1 and 1.5 mg/kg/day were simulated for a virtual population of 1000 patients, considering free drug plasma concentrations for a typical unbound fraction of 0.045 [27]. Additional simulations were performed to test scenarios where amphotericin B MICs for *C. auris* were 0.06–0.5 mg/L, according to the following equation [28]:

(6) $$\mathrm{MIC}={\left(\frac{d}{E_{\max}-d}\right)}^{1/h}\times {\mathrm{EC}}_{50}$$

where d is a drug-independent constant and h is the Hill factor. The EC_50_ value for each MIC scenario was then included in the PK/PD model and simulations were performed similarly. All simulations were conducted with NONMEM and S-PLUS.

## 3. Results

### 3.1. Time-Kill Experiments

**Figure 1:**
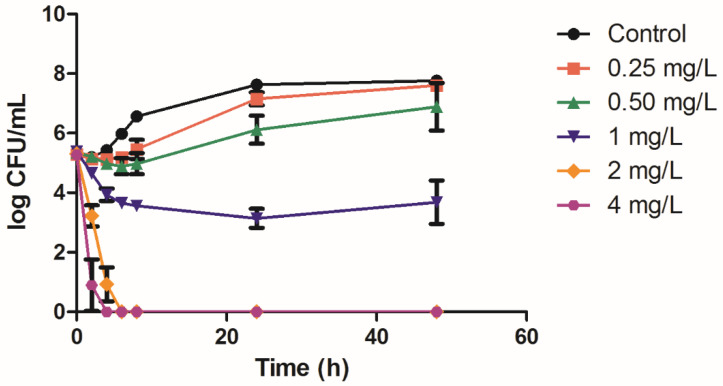
Mean time–kill curves for amphotericin B against *C. auris*. Each data point represents the mean result ± standard deviation (error bars) of the six isolates and replicates.

Graphical representation of mean T-K curves for all isolates and replicates is shown in Figure 1. No antifungal carryover was observed. Amphotericin B showed concentration-dependent fungicidal activity. Fungicidal effect (3 log reduction compared to initial inoculum) was rapidly achieved, at 2 and 4 h, for concentrations of 4 mg/L and 2 mg/L, respectively. At concentrations of 1 mg/mL (equal to MIC), the effect was fungistatic overall, with a biphasic killing kinetic trend that showed fungal regrowth by the end of the experiment in some clinical isolates.

### 3.2. Semi-Mechanistic PK/PD Modelling

**Scheme 1:**
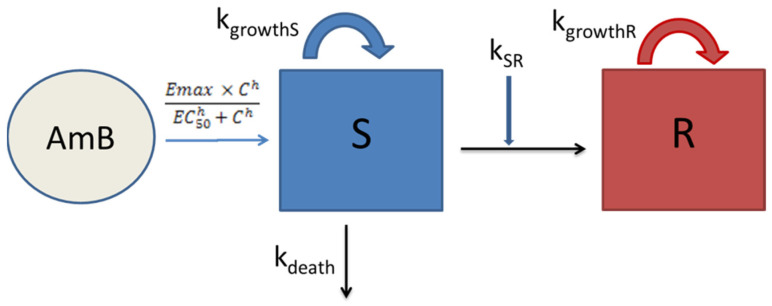
Schematic illustration of the final PK/PD model. The total fungal population consists of two different subpopulations (S + R), with a first-rate order constant (k_SR_) that describes the transfer of fungal cells from a susceptible state (S) to a resistant one (R). Amphotericin B (AMB) exerts its effect on the susceptible subpopulation. k_growthS_: growth-rate of susceptible subpopulation; k_growthR_: growth-rate of resistant subpopulation. k_death_: death-rate constant of the susceptible subpopulation.

The developed model was able to describe successfully the effect of amphotericin B against the studied *C. auris* clinical isolates. This model could characterize the initial and higher killing rate at the higher amphotericin B concentrations, 2 and 4 mg/L, as well as the biphasic trend or regrowth observed in most experiments with the concentration of 1 mg/L. A schematic illustration of the final model is shown in Scheme 1.

**Figure 2:**
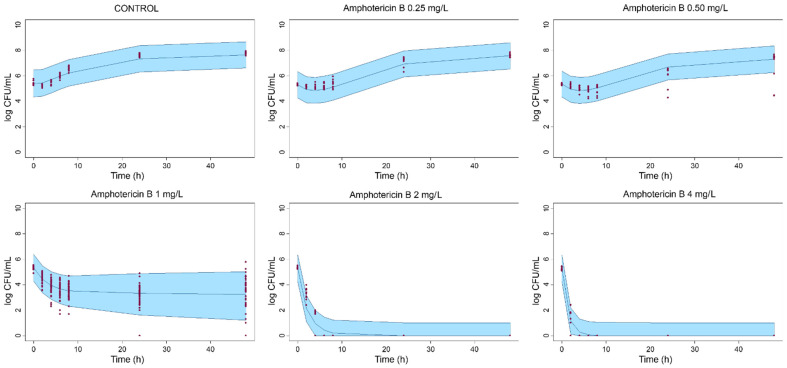
Visual predictive check (VPC) for the final model, with the observed fungal counts (full circles), the mean prediction (solid line) and 95% model prediction interval (shaded area) of the simulations.

Final model parameters and the standard error of the estimates, alongside bootstrap estimations are presented in Table 1. Considering the standard errors and the bootstrap results, the parameters of the model were properly estimated. *Candida* related parameters were k_growthS_ and k_death_ for S subpopulation (0.111 h^−1^ and 0.01 h^−1^, respectively) and k_growthR_ for R subpopulation (0.01 h^−1^). k_death_ and k_growthR_ were fixed whereas k_growthS_ was allowed to be estimated. When the model incorporated different values of α (delay in growth) for the absence or presence of the drug, a better fit was achieved. A modified E_max_ sigmoidal model best described the effect of the drug; E_max_ was equal to 0.784 h^−1^ and EC_50_ was equal to 1.88 mg/L (1.88 times bigger than the MIC). Hill factor was fixed to enable a proper estimation of the PD parameters. Variability in the response was best captured by IOV on EC_50_ rather than IIV, where each occasion (four in total) was defined as each prepared batch of microtitre plates. Model appropriateness was supported by the VPCs depicted in Figure 2.

### 3.3. Simulation of Standard Treatments Using Human PK Data

**Figure 3:**
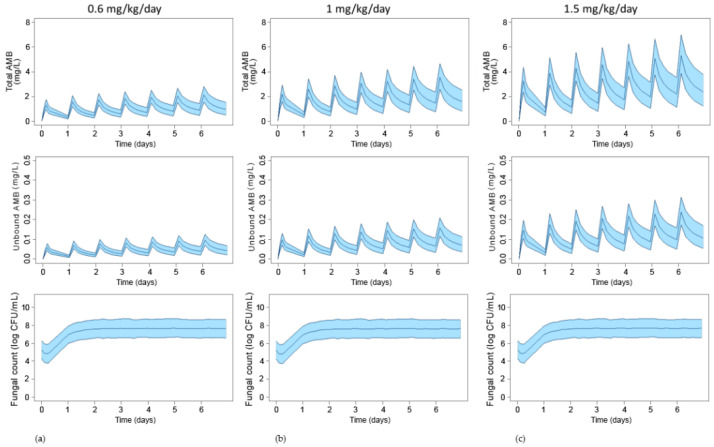
Predicted total (top row) and unbound (mid row) plasma concentrations of amphotericin B (D-AMB) and the effect on fungal burden (bottom row) for the treatment of 0.6 mg/kg/day (**a**), 1 mg/kg/day (**b**) and 1.5 mg/kg/day (**c**) of amphotericin B. The mean (solid line) and 95% prediction interval (coloured space) are represented.

The simulated total and unbound concentrations of amphotericin B for typical intravenous dosing regimens of 0.6, 1 and 1.5 mg/kg/day and their expected activity on *C. auris* after a one-week treatment are shown in Figure 3. None of the simulated standard dosing scenarios showed successful activity against *C. auris*.

**Figure 4:**
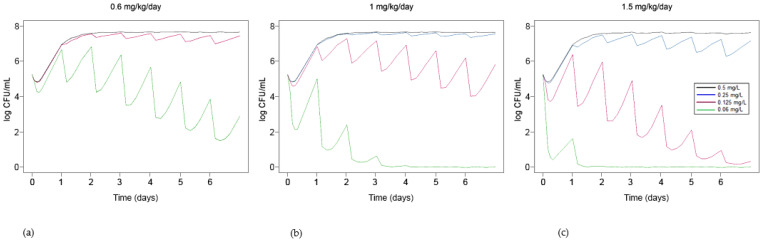
Simulations of the effect of amphotericin B on fungal burden by MIC and by dosing regimens of 0.6 mg/kg/day (**a**), 1 mg/kg/day (**b**) and 1.5 mg/kg/day (**c**). Black line: MIC of 0.5 mg/L; blue line: MIC of 0.25 mg/L; purple line: MIC of 0.125 mg/L; green line: MIC of 0.06 mg/L.

Additional simulations with MIC scenarios of 0.06, 0.125, 0.25 and 0.5 mg/L (with EC_50_ of 0.12, 0.24, 0.47 and 0.94 mg/L, respectively) for a 1-week period are presented in Figure 4.

Simulations with the lowest dose, 0.6 mg/kg/day, showed that a fungistatic activity would be achieved at the 5th day of treatment for MIC values of amphotericin B of 0.06 mg/L. The next simulated dose, 1 mg/kg/day, resulted in fungicidal activity from the second day onwards and fungistatic with the first administration. Finally, the highest dose of 1.5 mg/kg/day led to a fungicidal endpoint immediately after the first administration. Additionally, for an MIC of 0.125 mg/L a fungistatic effect would be achieved at the 3rd day, and fungicidal at the 5th day at this highest dose level.

## 4. Discussion

*C. auris* is an emergent fungal pathogen with reduced susceptibility to first-line antifungal agents. Amphotericin B is an antifungal drug with proven efficacy against invasive candidiasis and a low resistance-rate despite more than six decades of use; a correct and thorough knowledge about the activity of this drug against *C. auris* is necessary. To date, most susceptibility studies on this pathogen have focused on the determination of the MIC. The MIC is the standard PD parameter used as a marker of fungal susceptibility and antimicrobial efficacy, yet it possesses some limitations. The antimicrobial activity of drugs is a dynamic process, while MIC is a threshold value. Concentrations below or above the MIC are ignored and thus, a more precise and quantitative information about the concentration-effect profile of the drug is often missing [29]. It is also noteworthy that even for the same microbial species, same MIC values among different isolates can result in different killing kinetics [24]. Thus, studies that characterize the antimicrobial activity beyond the measurement of MIC are needed. In vitro time–kill curves allow obtaining more information about the effect of different drug concentrations on microbial population over a time period. In combination with PK/PD M&S, these time–kill curve experiments provide an interesting tool to predict and simulate untested scenarios that may help in decision-making and design of further studies.

As determined for other species of *Candida* [30,31], in the present study amphotericin B showed concentration-dependent activity against *C. auris* in T-K experiments. Fungicidal activity was achieved at concentrations ≥2 mg/L and in less than 2 h. These results are in agreement with the only published work based on T-K curve methodology against *C. auris*, in which MIC values of amphotericin B were also 1 mg/L [16]. Additionally, this killing kinetic pattern has also been described for other species of *Candida* that have been regarded as resistant to amphotericin B treatment [31,32].

Few PK/PD models are available for antifungal agents [19,20,21]. To our knowledge, the present study is the first work that used a semi-mechanistic approach to model the antifungal activity of amphotericin B against *C. auris*. The static T-K experiments performed showed fungal regrowth or a biphasic trend, and therefore, a semi-mechanistic model that included two fungal subpopulations with different susceptibility to the drug best captured this behaviour. This approach has been extensively applied to successfully model antibiotic activity [25,33]. In the model of the present study, the emergence of resistance is triggered by a high microbial count, with the susceptible population switching to a resistant one, a process described by a first-order rate constant that also accounted for the self-limiting growth rate, as it has been previously proposed by other authors [25,34]. The model best fitted the data when a different growth rate constant for the resistant subpopulation was defined (k_growthR_); this parameter was estimated to be 10 times lower than the growth rate constant for the susceptible subpopulation (k_growthS_), which is in agreement with the ‘fitness cost’ observed in some species of *Candida* when they develop resistance mechanisms [35]. Moreover, phenotypic switching during treatment with amphotericin B has been described for *Candida lusitaniae* [36], a closely related species of *C. auris*. Nevertheless, the main goal of model building was to accurately describe the antimicrobial activity and perform simulations rather than to provide insight into resistance mechanisms, for which specific microbiological and molecular procedures would be needed. On the other hand, the lack of similar PK/PD models reports for *C. auris* or amphotericin B in the literature has precluded comparisons.

A three-compartment model for amphotericin B deoxycholate [26] was implemented for the simulation of plasma concentrations of the drug in human patients for dosing regimens of 0.6, 1 and 1.5 mg/kg/day. The latter is more commonly used for invasive aspergillosis rather than for candidemia [37], but we considered this dosing schedule for simulation purposes too due to the low susceptibility profile of *C. auris* and the concentration-dependent PD of amphotericin B shown in the present study, in concordance with other in vitro and in vivo results [16,38]. Higher doses were not considered due to the toxicity of the drug. Amphotericin B, as many antifungal agents, is highly bounded to plasma proteins, around 95% at clinically achievable concentrations [27], and this feature was taken into account in the PK/PD simulations.

With regard to the different pharmaceutical formulations available, liposomal amphotericin B (L-AMB) is the current first choice due to its improved safety profile and comparable efficacy to conventional amphotericin B deoxycholate. However, the cost can be too high for healthcare systems in developing countries, which makes the conventional formulation still relevant and listed as essential drug [39]. On the other hand, it is still not clear which fraction of the total plasma concentration of L-AMB is active, as protein binding is not applicable for this formulation [40], which makes the bridging between in vitro experiments and in vivo simulations harder to perform. Therefore, the simulations were carried out for the deoxycholate formulation. Nevertheless, studies in animal models of invasive candidiasis and clinical trials have shown similar efficacy for both formulations [40]. This may reflect a comparable exposition of the fungal population to free drug and therefore conclusions driven by PK/PD simulations may be applicable for L-AMB too. In fact, EUCAST susceptibility breakpoints are based on adult standard dosages of both formulations [41].

As previously mentioned, an approach solely based on the MIC of antimicrobials provides limited information. Conversely, PD parameters derived from the analysis of T-K curves, such as E_max_ and EC_50,_ give more detailed information on the activity of the drug. However, obtaining these data in the clinical setting is time-consuming, laborious and usually not feasible. This drawback can be overcome by employing mathematical relationship between the MIC and EC_50_, as it has been demonstrated in the present study, since it is possible to link the results of the PK/PD modelling and simulation of T-K curves with the MIC of the drug [28].

Simulations of standard dosages of amphotericin B deoxycholate pointed out that the treatment would not be effective against the clinical isolates tested in this study. Additionally, other possible treatment outcomes were tested by simulating different susceptibility scenarios, with MICs below 1 mg/L. Standard treatments of 0.6 and 1 mg/kg/day would only be effective against *C. auris* isolates for an MIC of 0.06 mg/L. However, a higher dosage of 1.5 mg/kg/day would also be effective for an MIC up to 0.125 mg/L. Therefore, contrary to expectations [7], susceptibility breakpoint of amphotericin B for *C. auris* might be lower than 1 mg/L. Similar threshold values for amphotericin B have been reported for other species of *Candida* and filamentous fungi, such as *Aspergillus*. In a murine model of invasive candidiasis caused by *Candida krusei*, a daily dose of 1 mg/kg of amphotericin B was effective in reducing the kidney fungal burden when the MIC of the drug was of 0.125 mg/L, but ineffective when MIC was of 0.5 mg/L [42]. In another murine model study, doses of 1.5 mg/kg/day of amphotericin B resulted in a 15-day survival percentage of >50% for *Candida glabrata* and <25% for *Candida tropicalis*, the MIC being 1 mg/L for both species [43]. In an in vitro dynamic system that mimicked human PK of unbound amphotericin B against *Aspergillus*, those species considered resistant to amphotericin B had a probability of target attainment (PTA) of 0% when the MIC was 1 mg/L; for a PTA of 80% an MIC of 0.25 mg/L was needed [44]. On the other hand, a work that analysed the effect of antifungal drugs against *C. auris* infection in a murine model of invasive candidiasis concluded that the MIC cut-off for amphotericin B was 1.5 mg/L [38]. However, variability between strains was high and the 50% effective dose (ED_50_) was as high as 5 mg/kg/day, a dose that can be lethal [45].

The results obtained in this study should be cautiously interpreted, as in vitro-in vivo correlation studies for amphotericin B against *C. auris* are lacking. Even though T-K curve methodology is a more complex technique that provides further information than MIC determination, it is still an in vitro approximation to the much more complex in vivo reality. Factors such as host immunity status and drug tissue distribution are overlooked, whereas fungal burden may be overestimated, as growth rate is much faster in the rich environment of the microbiological broth culture than in the human infection sites [46]. Nevertheless, the developed model and simulation results may help in the design of future preclinical and clinical studies, providing a useful tool for dosing regimen selection. It would also be of interest to further confirm in a murine candidiasis model if the MIC of 1 mg/L is linked to treatment failure.

## 5. Conclusions

In conclusion, the developed PK/PD model was able to properly characterize the antifungal activity of amphotericin B against *C. auris.* The simulations highlighted that an MIC of 1 mg/L would be linked to treatment failure and in consequence, the amphotericin B resistance rate in this fungal species may be higher than previously reported [1]. These results may be extrapolated to *C. auris* clinical isolates with similar EC_50_/MIC ratio. Nevertheless, further studies are needed to fully characterize the susceptibility profile of *C. auris* and optimize antifungal therapy.

## Figures and Tables

**Table 1:** Parameter estimates (typical values and relative standard error –RSE– as CV %) and bootstrap estimates (mean and 95% CI) of the PK/PD model.

Parameter	Description	Model Estimate and RSE (CV %)	Bootstrap Estimate (Mean and 95% CI)
k_growthS_ (h^−1^)	Fungal growth rate constant of the S subpopulation	0.111 (3%)	0.111 (0.101–0.116)
k_growthR_ (h^−1^)	Fungal growth rate constant of the R subpopulation	0.01 (fixed)	-
k_death_ (h^−1^)	Fungal death rate constant	0.01 (fixed)	-
E_max_ (h^−1^)	Maximum kill rate constant of amphotericin B	0.784 (12%)	0.795 (0.635–1.04)
EC_50_ (mg/L)	Concentration of amphotericin B at which 50% of the E_max_ is achieved	1.88 (3%)	1.89 (1.78–2.05)
h	Hill factor that that modifies the steepness of the slope and smoothens the curve	4 (fixed)	-
α (control)	Delay in fungal growth in the absence of drug	0.748 (3%)	0.754 (0.664–0.882)
α (drug)	Delay in fungal growth in the presence of drug	0.231 (10%)	0.233 (0.193–0.274)
N_max_ (log CFU/mL)	Maximum fungal density	7.66 (1%)	7.67 (7.47–7.87)
σ (log CFU/mL)	Residual error	0.271 (14%)	0.270 (0.190–0.327)
π_1_ (%CV)	Occasion 1	0 (fixed)	-
π_2_ (%CV)	Occasion 2	9.5 (35%)	9.22 (2.45–15.34)
π_3_ (%CV)	Occasion 3	18.4 (24%)	18.76 (10.07–28.12)
π_4_ (%CV)	Occasion 4	7.5 (37%)	7.13 (2.75–13.19)
